# Sexual display complexity varies non-linearly with age and predicts breeding status in greater flamingos

**DOI:** 10.1038/srep36242

**Published:** 2016-11-24

**Authors:** Charlotte Perrot, Arnaud Béchet, Céline Hanzen, Antoine Arnaud, Roger Pradel, Frank Cézilly

**Affiliations:** 1 Institut de Recherche de la Tour du Valat, Arles, France; 2 CEFE UMR 5175, CNRS – Université de Montpellier – Université Paul-Valéry Montpellier – EPHE, Montpellier Cedex 05, France; 3 Université de Bourgogne-Franche Comté, UMR CNRS 6282 Biogéosciences, Dijon, France

## Abstract

The long-lived greater flamingo (*Phoenicopterus roseus*) is famous for performing conspicuous group displays during which adults try to acquire a new mate each year with varying success. We examined variation in the sexual display complexity (SDC) of wild flamingos aged between 4 and 37 yrs. SDC was defined as the product of richness (the number of different display movements) and versatility (the number of transitions between movements) within a 5 min behavioral sequence. In both sexes, date in the pairing season had a linear and positive effect on SDC, whereas age had a quadratic effect, with SDC increasing until about age 20yrs, and declining afterwards. SDC better explained pairing patterns than age, and positively influenced the probability of becoming a breeder. Our results thus support the idea that SDC is an honest signal of individual quality and further suggest that senescence in display could be an overlooked aspect of reproductive decline in species with no or weak pair bonding.

Various animal species, including both vertebrates and invertebrates, perform sexual displays. Such displays are considered to be complex (or elaborated) when they combine several different movements organized in a more or less stereotyped and repetitive sequence, in contrast with more simple displays consisting of only one or a few single movements^1^,^2^. For instance, the relatively simple display of male magnificent frigatebirds, *Fregata magnificens*, consists in outstretching and vibrating wings rapidly with head thrown back, and red gular pouch fully blown out^3^. In contrast, males of several *Anas* duck species typically perform complex, highly ritualized sexual displays that include several distinct motor patterns organized in a fixed and ordered sequence^4^,^5^.

Due to the energy and time cost associated with their production, complex displays have often been regarded as honest signals of individual quality^6^. For instance, in several lek-breeding bird species, males perform complex sexual display involving different movements such as wing beats, foot stamping and high jumps^7^, with displays effort predicting male mating success^8^,^9^. However, the energetic cost of a display may not necessarily be related to its complexity. For instance, a display can consist of a single movement that is performed repeatedly^2^, such that it is energetically costly but not particularly complex. Therefore, some particular benefits must be associated with complexity *per se*. Accordingly, various benefits of complex signaling have been suggested, independently of its energetic cost^1^. For instance, different components of a complex display may signal different aspects of an individual’s quality^10^,^11^ or work as different signals that serve to match the variable preferences of different receivers^12^. Alternatively, complexity in display might be a form of redundancy (i.e. the different components convey the same information) that allows for an increased accuracy of the receiver response^10^,^11^,^13^, or a strategy to cope with the variable transmission and reception efficiency of different signals across different environments^14^,^15^.

Although each of these interpretations have received some empirical support^1^, little is known about the causes and consequences of inter-individual variation in the complexity of “complex” sexual displays (but see ref. 16). So far, quantitative studies of sexual display complexity (SDC) have examined co-variation between the multiple components of the display and the signaling value of each one (e.g.^17^,^18^). To the best of our knowledge, no empirical study to date has quantified SDC and related it to individual characteristics or fitness consequences. This might be due to the recurring difficulty in defining and quantifying complexity in biology^19^ (but see ref. 20), particularly when considering multimodal displays. In addition, most, if not all, studies of complex sexual displays concern species where one sex (usually the male) is displaying to the other one (usually the female)^21^,^22^,^23^,^24^. However, complex displays can also be observed in species with mutual mate choice, particularly among socially monogamous bird species^25^,^26^,^27^,^28^,^29^.

It has been further suggested that large and dense social groups demand more complexity in signaling because of the need to transmit information to a large number of individuals^20^. In that respect, flamingos (Phoenicopteridae) might be a particularly well-suited species to investigate inter-individual variation in SDC. Flamingos are obligate colonial breeders, and perform conspicuous mixed-sex group (or communal) displays^30^, which are supposed to stimulate synchronous breeding and facilitate pair formation. Up to several thousand individuals then form dense aggregations and perform in synchrony a variety of movements in a more or less stereotyped succession for several hours per day during the pre-breeding period^26^,^31^,^32^,^33^. The group display of flamingos is therefore a good example of “communicative complexity” (*sensu*^20^), as it contains several structurally distinct ritualized elements^26^,^31^. Still, the display repertoire of flamingos remains limited in size, such that it can be reasonably used to quantify SDC^34^, defined here as the product of display richness (i.e. the number of different acts in a sequence) by display versatility (i.e. the number of transitions between acts in a sequence).

Using a cross-sectional approach, we investigated inter-individual variation in SDC in the greater flamingo, *Phoenicopterus roseus*, taking advantage of a long-term study that today results in the observation in the Camargue (southern France) of on average 3000 individually marked birds of known sex and age each year^26^. Although the species is socially monogamous, it is characterized by the total absence of long-term pair bonding, with all pairs divorcing systematically between two consecutive breeding seasons^35^. This means that all sexually mature individuals must invest each year in group displays in order to find a new mate and be able to breed. However, some birds succeed to acquire mates whilst others remain unpaired. Most of the plumage of greater flamingos is pale pink but during group displays, they typically exhibit their underwing colorations which offer a bright contrast between carotenoid-based crimson remiges and melanin-based black ones (Fig. 1). Carotenoid-based plumage is dependent on diet (as birds cannot synthesize carotenoids *de novo*), and is thought to reflect current condition, whereas melanin-based plumage is synthesized as a by-product of amino-acid catabolism, is under genetic control, and is likely to reflect genetic quality of individuals^1^. In addition, such ornaments may act to enhance the apparent skill and vigor of individual motor performance during group displays^6^. We provide for the first time evidence for a quadratic age effect on SDC, suggestive of early improvement and senescence, assortative mating for SDC among pairs of greater flamingos, and a positive influence of SDC on the probability of becoming a breeding individual.

## Results

The age of the focal individuals ranged from 4 to 34 years for males and from 5 to 37 for females. Within a five-minute courtship sequence, the number of postures varied from 2 to 8, while the number of transitions between postures (versatility) varied between 2 and 17. SDC scores consequently varied from 4 to 136.

**Figure 2:**
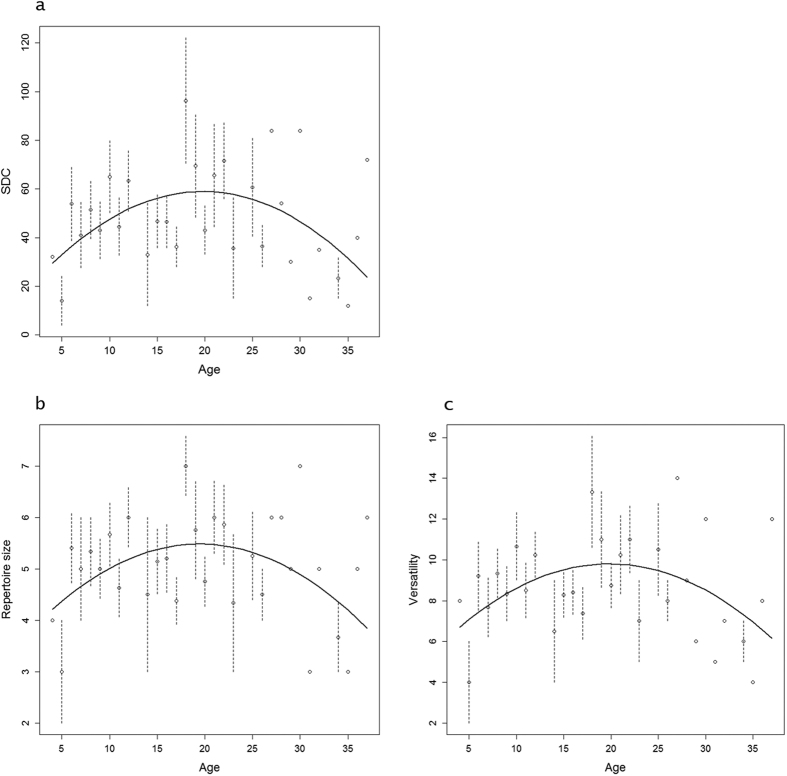
Quadratic relationship between age and (**a**) SDC (**b**) courtship repertoire size (**c**) courtship versatility of individuals in the greater flamingo according to model 1 (age + age^2^ + date) for the tree variable, with date fixed at February 3. Individual points correspond to the arithmetic mean of observed SDC, repertoire size or versatility per age ± SE when there was more than 1 observation.

Three models explained SDC almost equally well (Table 1). Model averaging indicated a quadratic effect of age on SDC (Table 2), with SDC being higher in individuals of intermediate age compared to young and old ones. For instance, SDC was 1.7 times higher in 20-year-old individuals compared to 6 and 34 year-old individuals (Fig. 2). SDC increased over the courtship season (Table 2), but was not influenced by either sex or year. In the same way, quadratic effects of age and date of courtship display were also retained in the best models explaining variability of repertoire size and versatility of sexual display, and model averaging indicated both higher repertoire size and higher versatility in individuals of intermediate age compared to young and old ones. Repertoire size was 1.2 times higher in 20-year-old individuals compared to 6 and 34 year-old individuals and versatility was 1.3 times higher in 20-year-old individuals compared to 6 and 34 year-old individuals (Fig. 2b,c). As for SDC, repertoire size and versatility increased throughout the courtship season (Table 2).

**Figure 3:**
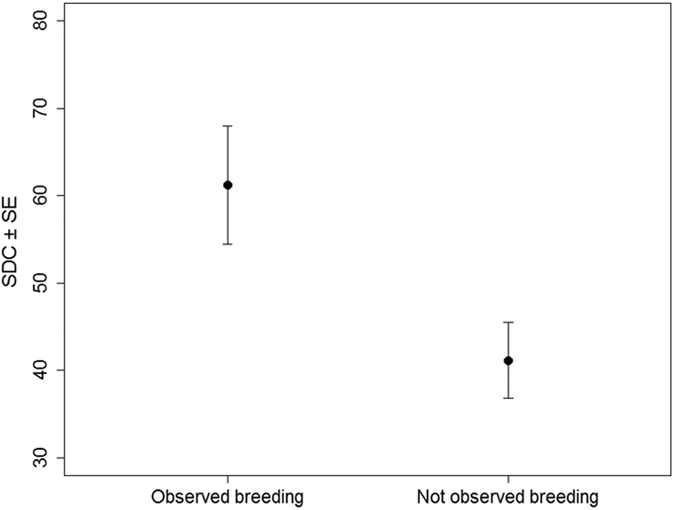
Mean (± SE) SDC of individuals confirmed (43) and not confirmed (13) as breeders at the breeding colony in the year 2015.

SDC was a good predictor of the future breeding status of individuals (Wilcoxon–Mann–Whitney test: W = 146.5, *p* = 0.001), with confirmed breeders having a mean SDC score of 61.23 (±6.76 SE) compared to a mean SDC score of 41.14 (±4.33 SE) in individuals not confirmed as breeders (Fig. 3). In addition, SDC was retained in the three best models explaining the probability that an individual was observed at the colony (Table 1). Model averaging indicated a positive influence of SDC on breeding status (Table 2). There was no effect of sex or age on breeding status after accounting for SDC.

**Figure 4:**
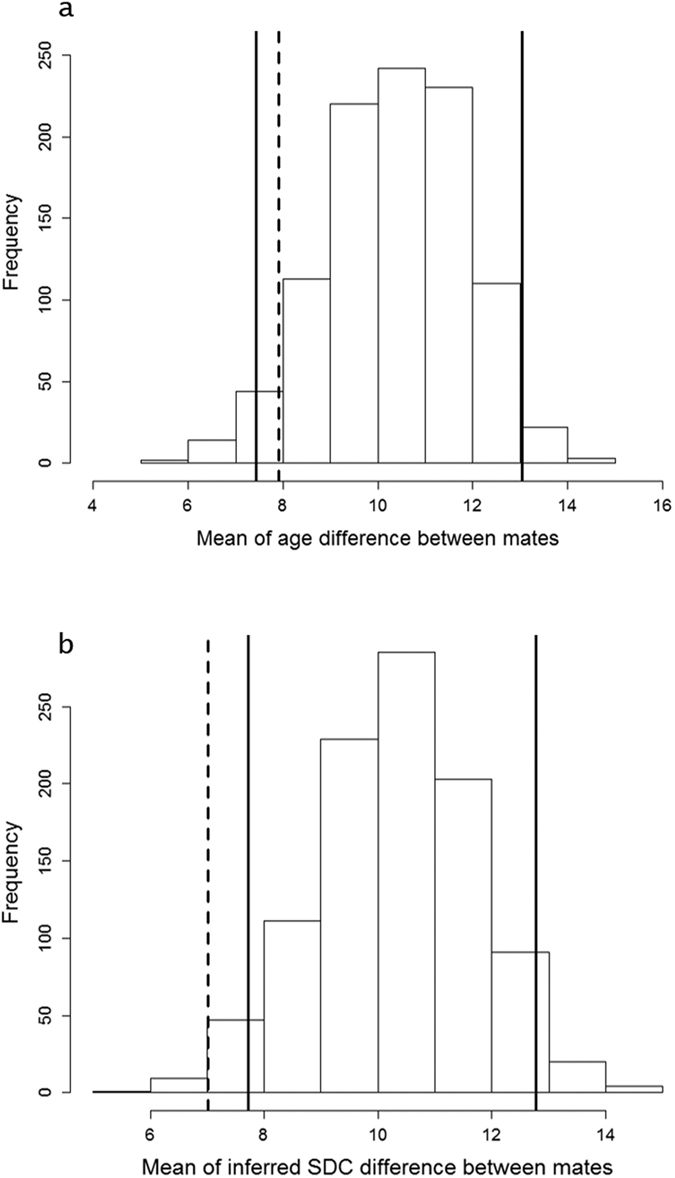
(**a**) Distribution of mean age difference under the assumption of random pairing with respect to age. Full lines correspond to the upper and lower 95% confidence limits, dashed line corresponds to the observed mean of age difference between mates in our sample of flamingo pairs. (**b**) Distribution of mean SDC differences under the assumption of random pairing with respect to complexity. SDC values were inferred from age according to the relationship SDC ~ age + age^2^ + date (model 1, Table 1). Full lines correspond to the upper and lower 95% confidence limits, dashed line corresponds to the mean of inferred SDC difference between mates in our sample of flamingo pairs.

In 2015, the age difference between mates ranged between 0 and 27 yrs, with a mean value of 7.905 (±1.793 SE; n = 21) yrs. There was no evidence for males being older than females in pair, or the reverse (Wilcoxon matched-pairs test: W = 115.5, *p* = 0.708). The observed mean age difference between mates was within the confidence interval of the simulated distribution based on random mating (μ_obs_ = 7.905; 95%CI_distribution_ = [7.429; 13.048]; Fig. 4a), indicating that age did not significantly affect pairing patterns. In contrast, the observed mean difference in age-related SDC between mates lied outside of the confidence interval of the simulated distribution based on random mating (μ_obs_ = 7.020; 95%CI_distribution_ = [7.721, 12.779], Fig. 4b), thus suggesting homogamy for SDC.

## Discussion

Our study provides strong support for the hypothesis that SDC *per se* is an honest signal of individual quality involved in mate choice^1^ in the greater flamingo. SDC, defined here as the product of display richness by display versatility, varied extensively between individual flamingos, with no difference between sexes, and this variation was partly explained by variation in both the date of observation and the age of individuals. In turn, SDC positively influenced the probability of becoming a breeding individual, being about 50% higher in confirmed breeders *vs*. not confirmed breeders. Taken together, our results suggest that high SDC in greater flamingos signals high individual quality and current vigor^6^, and, hence, superior competitive ability to secure a nest site on a crowded breeding island where access to nesting space is very limited^22^. SDC in flamingos could then play a role analogous to song complexity in songbirds, where males with high song complexity have been shown to obtain high quality territories and be more efficient at defending them^24^.

The signal value of SDC and its energy cost may further explain the observed increase in SDC through time, independently of age. Although high quality individuals might be able to pay the full energy cost of complex displays early in the pairing season, lower quality individuals might not be able to perform costly complex displays, particularly at the beginning of the pairing season, between November and January, when temperatures are at the lowest in the Camargue (average temperatures of 9.4 °C with minima near 1 °C). However, towards the end of the winter, as temperatures increase (average temperature of 11.2 °C with minima near 7 °C in March 2015) individuals still unpaired may ultimately increase their display effort in a final attempt to attract a breeding partner. This interpretation does not rule out other phenomena, such as an increase in hormone levels due to sustained social stimulation^32^. The existence of such a mechanism could be investigated in captive flocks of flamingos.

The fact that age-related variation in SDC better explains the observed pairing patterns than age itself further reinforces the idea that SDC signals individual quality. As incubation and chick provisioning duties are equally shared between the male and the female in pairs of greater flamingos, mutual mate choice for quality is expected, thus leading to assortative mating for quality as reflected in SDC. Age-assortative mating had previously been reported in the Camargue population of greater flamingos^36^, contrasting with the present results. However, in that earlier study, the age of individuals ranged only from 3 to 15 yrs, whereas in the present one the age of paired birds that were ringed varied between 4 and 37 yrs. The pattern of age-assortative mating previously reported could then simply result from the positive association between age and SDC in younger age classes. Our results show that very old individuals can actually be paired with young ones, as they happen to be similar in terms of display complexity.

Our most important result may however lie in the observed quadratic effect of age on SDC, suggestive of improved motor performance with increasing age, followed by a period of senescence. This is, to the best of our knowledge, the first evidence for senescence affecting a sexual motor display. It is however in accordance with some previous results on the relationship between age and sexual display. For instance, a quadratic relationship between lek attendance (but not fighting rate or distance to the center of the lek) and age has been reported in male black grouse^37^, while a concave relationship between song consistency (but not repertoire size) and age has been observed in a free-living population of great tits, *Parus major*^38^. In the present study, a quadratic effect of age was detected on both repertoire size and the number of transitions between movements, indicating that both components of motor display are affected by senescence.

The observed increase in SDC during early life suggests that flamingos may acquire their motor competences progressively through a maturation process^39^. However, this result is based on a cross-sectional study, with each individual having been sampled only once, in one of two consecutive breeding seasons. As within-individual variation has not been taken into account, the observed pattern could have been generated by the disappearance of poor quality individuals and/or the appearance of high-quality individuals with age^40^. A longitudinal study of SDC, involving the collection of repeated display sequences of the same individual at different ages, would be necessary to discriminate between the two processes. However, this approach would not be easy to apply, as the collection of field data across several years would be labor intensive, and the probability of re-observing the same individuals over multiple seasons rather low, given the high dispersal of flamingos between breeding colonies in the Mediterranean region and their irregular breeding at that regional scale^41^. Such an approach might however be possible using captive flocks of flamingos.

Symmetrically, the observed decrease in SDC after age 20 likely reflects reproductive senescence in wild flamingos. Alternatively, old birds might be more experienced at successfully acquiring a reproductive partner, and a lower SDC could simply correspond to a modulation of their investment in sexual display^42^. However, this explanation is unlikely as the probability to become a breeder increased with increasing SDC for both males and females. On the other hand, competing for mates may incur substantial costs, particularly when individuals need to invest heavily in the production of sexual signals to attract a reproductive partner. As engaging in group displays must be energetically demanding in flamingos (as it has been shown in other bird species^37^,^38^,^43^), it may increase metabolic rate and, hence the production of reactive oxygen species that can damage biomolecules, unless regulated by enzymatic and non-enzymatic antioxidant systems^44^. Interestingly, a quadratic age effect has been found in resistance to oxidative stress in a captive population of greater flamingos^45^. Similar to SDC in the present study, resistance to oxidative stress increased for age between 0 and 15yrs to reach an asymptote between 16 and 25yrs, and finally slightly decreased at older ages. Oxidative stress may then limit display effort in wild greater flamingos and could explain the observed quadratic relationship between SDC and age observed in the present study.

Previous studies on greater flamingos in the wild reported an increase in survival, breeding propensity and breeding success with age, but failed to detect any pattern of senescence^46^,^47^,^48^. However, the maximum age of individuals included in these studies was 20 yrs. Still, as flamingos divorce each year^26^, senescence may actually take place early in the reproductive season, at the time of pairing and acts as a filter. More precisely, only the best individuals among the older ones could manage to find a partner and consequently, senescence might not be detected afterwards. Our results thus suggest that the influence of the dynamics of pair bonding and that of costly and complex sexual displays on patterns of reproductive senescence in the wild deserve further consideration.

## Methods

### Behavioral observations

Observations were made in the Camargue, Southern France, one of the most important breeding sites of greater flamingos in the Mediterranean region^49^, during two consecutive seasons of courtship displays (November to March in both 2014 and 2015). Since 1977, on average, 12% (7–30%) of the chicks fledged in the Camargue have been marked with PVC plastic rings engraved with a three or four digit alphanumerical code^50^, allowing individual identification at distance and providing information about the age of individuals. In addition, the sex of ringed birds has been regularly ascertained, through behavioral observations or through blood sampling and molecular analyses^51^. Ringing and sample collection of greater flamingo chicks were authorized through the personal permit (number 405) of Alan Johnson and Arnaud Béchet delivered by the Centre de Recherche sur la Biologie des Populations d’Oiseaux (CRBPO, Muséum national d’histoire naturelle, France).

**Figure 5:**
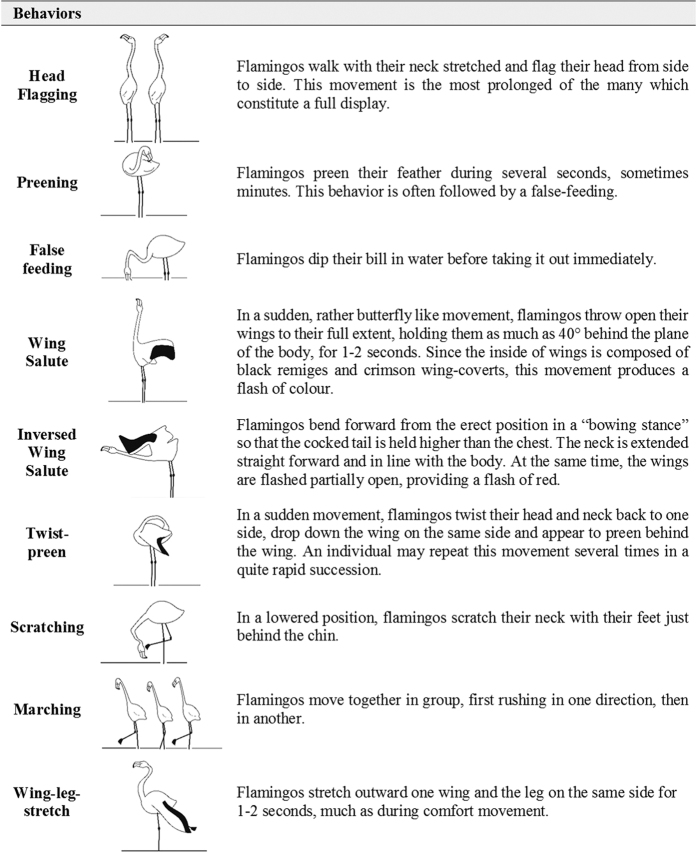
Behavioral repertoire of SDC in the greater flamingo derived from Johnson and Cézilly (2007). (Drawings by Samuel Hilaire).

Using a FullHD video camera equipped with a 60x zoom (20–1200 mm, Panasonic Lumix FZ72), we recorded the behavior of ringed individuals during displays. On each occasion, we attempted to follow a single displaying individual for up to five minutes. To that end, we first located a display group at a distance of less than 300 meters, at which the code engraved on a flamingo ring is readable^26^ and at which good quality videos can be recorded. We then looked for a ringed individual displaying within the courtship group and started recording its behavior. In addition, we recorded, for each individual sequence, the size of the display group (ranged from 9 to 130 individuals), the hour of the day and the date (as such variables were previously found to influence display behavior in flamingos^26^). However, many observations were interrupted before that time due to movements of individuals, agonistic interactions, or because the focal bird stopped displaying. We thus randomly selected 100 focal-individual sequences of different individuals (50 females, 50 males) where the display behavior had been recorded continuously for five minutes. Behavioral sequences were then coded using the JWatcher software^52^ in order to estimate courtship complexity. Following previous studies of the display repertoire of the greater flamingo^26^, nine different postures were recognized (Fig. 5).

### Display complexity

We analyzed sexual displays of greater flamingos as sequences of discrete postures from a finite repertoire, and relied on a simple method, widely used in the study of bird song, to assess complexity. Following^53^,^54^,^55^, we defined sexual display richness as the number of different postures in a sequence (i.e. repertoire size), and versatility as the number of transitions between different postures in a sequence. SDC was then calculated as the product of display richness and display versatility. Thus, complex sexual displays correspond to sequences where numerous transitions occur between a maximum number of postures, whereas simple ones correspond to monotonous sequences with high continuity and low versatility.

### Statistical analyses

We first investigated if the age and sex of individuals had an influence on the variability of display complexity, repertoire size and versatility, using generalized linear models. The complete model contained the interaction between sex and age, the quadratic effect of age (thus testing for a potential effect of senescence), as well as group size, hour of the day, date of the year, year and the interaction between year and date as explanatory variables. Model assumptions (i.e. normality and homoscedasticity of residuals) were checked. From the complete model we derived a set of all possible submodels. As group size and date were significantly correlated (r = −0.475, 95%CI = −0.643; −0.274), we removed models containing both variables from the set of models to avoid collinearity.

To investigate the influence of courtship complexity on the subsequent breeding probability we assigned a score to each individual according to their observed reproductive status at the Fangassier breeding colony in the Camargue. An individual was considered to have been breeding if it was seen at the same place on the breeding island for at least 48 hours, or if it was seen with an egg or rearing a chick (see^26^ for details). Any flamingo not seen at the colony, or seen at the colony but not in one of the previously described states, was considered as a non-breeding individual. The analysis was restricted to the 2015 data set, because flamingos bred at a different location in 2014, where continuous monitoring was not possible. First we compared SDC between non-breeding (N = 43) and breeding individuals (N = 13), using a two-sided Wilcoxon–Mann–Whitney test. Next, we used generalized linear models with a binomial distribution to test the influence of courtship complexity on breeding probability. The complete model contained courtship complexity, sex, age and the quadratic effect of age as explanatory variables. Model assumptions (i.e. normality and homoscedasticity of residuals) were checked.

Following recent recommendations to produce model estimates comparable between and within studies^56^,^57^, we standardized all explanatory variables by centering and dividing by two standard deviations using the *arm* package^58^. To prevent overparameterization, we respected the sample size rule-of thumb of 10: 1 subjects to predictors in multiple regression^59^. Model selection was based on Akaike Information Criterion corrected for small sample size (AICc)^60^. When several models were within a ΔAICc of 2 from the best model, we employed a model averaging approach, using the so-called zero method^60^ implemented in the *MuMIn* package of R^61^ on models within two points of AICc from the best one. This allowed us to account for model selection uncertainty in order to obtain robust parameter estimates^57^.

Analyses on assortative mating were performed on a sample of pairs (N = 21) with both partners ringed observed in 2015. We first examined age-assortative mating using the absolute value of age difference between members of the same pair. We examined where the observed mean age difference was situated within its theoretical distribution under the assumption of random pairing with respect to age (1000 simulations). As courtship complexity had not been measured on the same individuals, we used the predicted values of complexity according to age from the previous model to allocate a score of complexity to each individual. We then relied on the same procedure to test for assortative mating for courtship complexity.

All analyses were conducted with R 3.0.3^62^.

## Additional Information

**How to cite this article**: Perrot, C. *et al.* Sexual display complexity varies non-linearly with age and predicts breeding status in greater flamingos. *Sci. Rep.* **6**, 36242; doi: 10.1038/srep36242 (2016).

**Publisher’s note:** Springer Nature remains neutral with regard to jurisdictional claims in published maps and institutional affiliations.

## Figures and Tables

**Figure 1:**
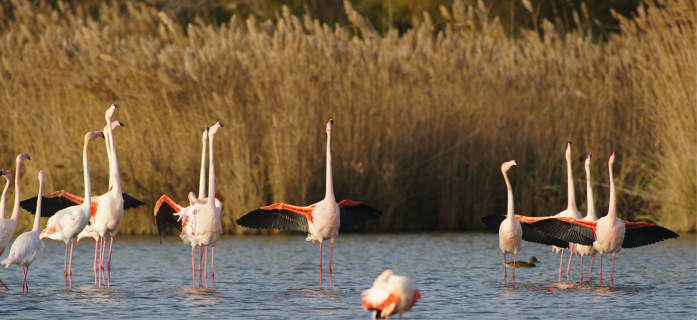
Group displays of greater flamingos in the Camargue (Photography by Benjamin Vollot).

**Table 1:** Model selection of the factors influencing sexual display complexity (SDC), repertoire size, and versatility of sexual display and the subsequent probability of observation at the breeding colony in the greater flamingo: age, age^2^, date, sex, group size, hour and year were tested on SDC, repertoire size and versatility; age, age^2^, SDC, and sex on probability of observation at the colony.

Response variable	Models	df	logLik	AICc	ΔAICc	weight
SDC						
	Age + age^2^ + date	5	−479.75	970.14	0	0.52
	Age + age^2^ + date + sex	6	−479.27	971.44	1.3	0.27
	Age + age^2^ + date + year	6	−479.57	971.44	1.9	0.20
	Date	3	−483.84	973.93	3.79	
	Age + date	4	−483.81	976.05	5.91	
	Age + age^2^	4	−485.29	979.00	8.86	
Repertoire size						
	Age + age^2^ + date	5	−177.51	365.67	0	0.57
	Age + age^2^ + date + sex	6	−177.31	367.52	1.85	0.22
	Age + age^2^ + date + age: sex	7	−176.21	367.65	1.98	0.21
	Date	3	−180.97	368.18	2.52	
	Age + date	4	−180.97	370.35	4.69	
	Age + age^2^	4	−182.54	373.50	7.83	
Versatility						
	Age + age^2^ + date	5	−260.95	532.53	0	0.66
	Age + age^2^ + date + sex	6	−260.46	533.82	1.29	0.34
	Date	3	−264.50	535.25	2.72	
	Age + date	4	−264.47	537.36	4.94	
	Age + age^2^	4	−265.53	539.48	6.95	
Probability of observation at the colony						
	SDC	2	−27.96	60.15	0	0.45
	SDC + sex	3	−27.15	60.76	0.61	0.33
	SDC + age	4	−27.52	61.50	1.35	0.23

Models with a ΔAICc ≤ 2 from the best model are represented. For sexual display variables, models under the dashed line are shown for comparison with the best model (SDC ~ age + age^2^ + date) for a better view of the strength of age, age^2^ and date effects.

**Table 2:** Model-averaged estimates ± SE and 95%CI of parameters explaining variations in SDC, repertoire size and versatility of sexual display and probability of observation at the colony in greater flamingos.

Response variable	Parameters	Estimate	SE	Confidence interval	Sum of weights
SDC					
	Intercept	58.097	3.981	(50.195; 65.998)	
	Age	9.778	6.749	(−3.62; 23.175)	1
	Age^2^	−29.926	10.591	(−50.948; −8.903)	1
	Date	20.623	6.559	(7.606; 33.638)	1
	Sex	−1.581	4.062	(−9.604; 6.442)	0.27
	Year	−0.836	3.546	(−7.856; 6.184)	0.20
Repertoire size					
	Intercept	5.449	0.194	(5.064; 5.833)	
	Age	0.385	0.328	(−0.267; 1.036)	1
	Age^2^	−1.318	0.519	(−2.347; −0.288)	1
	Date	0.988	0.308	(0.377; 1.598)	1
	Sex	−0.079	0.212	(−0.499; 0.342)	0.43
	Age: sex	0.185	0.456	(−0.712; 1.083)	0.21
Versatility					
	Intercept	9.718	0.445	(8.834; 10.602)	
	Age	1.035	0.756	(−0.466; 2.536)	1
	Age^2^	−3.144	1.182	(−5.490; −0.797)	1
	Date	2.138	0.705	(0.739; 3.537)	1
	Sex	−0.883	0.673	(−1.213; 0.767)	0.34
probability of observation at the colony					
	Intercept	−1.334	0.354	(−2.042 ; −0.623)	
	SDC	1.418	0.658	(0.099; 2.736)	1
	Age	0.145	0.419	(−0.687; 0.978)	0.33
	Sex	0.289	0.579	(−0.860; 1.436)	0.23

The relative importance of each factor is calculated by summing the AIC weights across the top models (Table 2) where the given factor appears (last column).
